# Influence of extent of surgical resection on post-hepatectomy shoulder pain: an observational study

**DOI:** 10.1038/s41598-023-38052-6

**Published:** 2023-07-05

**Authors:** Yuecheng Yang, Yunkui Zhang, ShengLing Dai, Lu Wang, Jun Zhang

**Affiliations:** 1 Department of Anesthesiology, Fudan University Shanghai Cancer Center, No 270, Dong-An Road, Shanghai, 200032 People’s Republic of China; 2 Department of Hepatic Surgery, Fudan University Shanghai Cancer Center, Shanghai, People’s Republic of China

**Keywords:** Diseases, Medical research

## Abstract

Shoulder pain frequently follows hepatectomy. However, the influence of surgical procedures on shoulder pain is unclear. In this observational study, patients who underwent hepatectomy were enrolled in Shanghai Cancer Center. Shoulder pain and surgical pain were assessed using the numeric rating scale 2 days after surgery. The incidence of shoulder pain was the outcome of the cohort study. Nested case–control analyses were further applied. Three hundred and twelve patients were finally enrolled in this study. Nested case–control analysis showed that there were no significant differences in the number of surgical segments between the two groups (*P* = 0.09). In addition, minor hepatectomy did not reduce the incidence of shoulder pain compared with major hepatectomy (*P* = 0.37). The drainage volume within 2 days after surgery was significantly more in those patients with shoulder pain (*P* = 0.017). In open surgery, surgical sites involving the right anterior lobe (OR (95% CI) 2.021 (1.075, 3.802), *P* = 0.029) and right posterior lobe (OR (95% CI) 2.322 (1.193, 4.522), *P* = 0.013) were both independent risk factors for shoulder pain. Left shoulder pain also occurred in patients who did not receive left lateral hepatectomy. The preventive phrenic nerve block was not suitable for post-hepatectomy shoulder pain. Stronger preventative intervention should be used in those high-risk patients.

## Introduction

Shoulder pain occurs frequently after both open and laparoscopic hepatectomy, which affects the life quality of patients. Post-operative shoulder pain was also associated with respiratory dysfunction^1^. The acknowledged mechanism of postoperative shoulder pain is the referred pain resulting from C3–5 nerve roots via the phrenic nerve^2^. The efficacy of phrenic nerve block (PNB) verified this hypothesis^3^. Previous studies found that younger age and the application of epidural anesthesia were the independent risk factors for post-hepatectomy shoulder pain (PHSP)^4,5^. However, few studies focused on the relationship between surgical procedures and PHSP.

As the liver clings to the diaphragm, surgical procedures may irritate the diaphragm. Hepatectomy is a non-standard surgery. Because of the different sizes and locations, tumors require different types of surgical procedures. The right lobe of the liver clings closely to the diaphragm, whereas the left lateral lobe of the liver is relatively dissociative. Previous studies found that mechanical stimulation (such as catheter and suture), endometriosis, and liver abscess around the diaphragm may cause ipsilateral shoulder pain^3,6–8^. The location of surgical sites is likely involved in the incidence of PHSP. Furthermore, in liver metastases, scattered tumors often require the resection of multiple segments. To the best of our knowledge, the relationship between the location and number of surgical segments and PHSP has not been investigated.

To fill the gap, we performed this observational study to explore the relationship between surgical liver segments and PHSP. This study may be helpful for the prediction and prevention of PHSP.

## Methods

### Study design

This prospective observational cohort study was conducted between June 2022 and November 2022. A nested case–control analysis was used to investigate the effect of different liver lobes on the incidence of shoulder pain. This study was approved by the Ethics Committee of Shanghai Cancer Center (No. 1612167-18). All patients signed informed consent. All research was performed in accordance with relevant guidelines.

### Patients

All patients underwent hepatectomy at Shanghai Cancer Center. No patients reported shoulder pain as the first symptom at clinic visits.

The inclusion criteria were: (1) Patients who underwent hepatectomy under anesthesia, (2) The American Society of Anesthesiologists Score of I–III, and (3) patients who denied a history of shoulder pain.

The exclusion criteria were: (1) persistent use of opioids, (2) multiple organ excision during the surgery, (3) surgery records cannot be recognized, and (4) laparoscopic surgery was changed to open surgery.

### Anesthesia and surgical procedures

The protocols of anesthesia and analgesia were determined by attending anesthesiologists. For patients who received epidural anesthesia, an epidural puncture was performed between T6 and T8. A test dose (3 ml of 1% lidocaine) was injected after the epidural catheter was placed. Epidural anesthesia was maintained using 0.25% ropivacaine. For all patients, general anesthesia was induced with propofol, rocuronium, and sufentanil, and maintained with inhaled anesthetics (sevoflurane or desflurane), propofol, and remifentanil. Sufentanil and rocuronium were injected intermittently during the surgery.

For patients receiving epidural anesthesia, patient-control epidural analgesia was provided. The formula of epidural analgesia was ropivacaine 300 mg combined with sufentanil 100ug. For patients receiving general anesthesia alone, the formula of intravenous analgesic was sufentanil 100ug combined with flurbiprofen 150 mg (or ketorolac tromethamine 90–105 mg). The total volume of the postoperative analgesia bag was 230 ml. The setting of the analgesia device was as follows: a background infusion of 4 ml/h, a bonus of 4 ml with a lockout time of 20 min.

All surgeries were completed by the same group of surgeons. The chief surgeons have more than 10 years of experience in hepatic surgeries. The patient was placed in the supine position if the surgical site was the left lobe of the liver. In the patient whose surgical site was in the right lobe of the liver, a padded cushion was placed under the patient’s waist to provide better exposure to the operative field. Both arms of the patient clung to the body during the surgery. In open surgeries, all incisions were made below the right costal margin. Retractors (Omni-Tract) were used to expose the operative field (Supplementary Fig. S1). In laparoscopic surgery, pneumoperitoneum was established and maintained at 10–12 mmHg. The intercostal port was not used in our institution. The drainage tube was placed in the cavity of the remaining liver. The tube did not contact with the diaphragm. In some major hepatectomies, another drainage tube was placed near the first porta hepatis.

### Data collection

Patients’ data were searched through electronic medical records. Age, gender, BMI, and perioperative data (including the use of laparoscopes, the use of epidural anesthesia, surgical duration, surgical position, bile leakage, and drainage volume within 2 days after surgery) were recorded. The surgical records were reviewed by two researchers independently. A surgical specialist was consulted when a difference of opinion arose. The number and the locations of surgical liver segments were recorded. Minor hepatectomy was defined as the resection of less than two liver segments. Shoulder pain and postoperative pain were assessed using a numeric rating scale (NRS) 2 days after the surgery. The investigator (DSL) who assessed the pain intensity was independent of anesthesia management, surgical procedures, and statistical treatment. The peak pain within 2 days (not the time the patient was asked) was recorded. The site of shoulder pain was also recorded. Moderate to severe pain was defined as a pain score ≥ 4.

### Cases and controls

In the cohort, cases were the patients who felt shoulder pain within 2 days after surgery. Controls were the patients denied any soreness or pain around the shoulder.

### Sample size calculation

The sample size was calculated using the software PASS (version 2021, USA). According to a previous study, the incidence of shoulder pain was 40%^4^. We assumed that the incidence of shoulder pain after minor hepatectomy and major hepatectomy was 30% and 50%, respectively. The numbers of the two groups were roughly assumed to be equal. The two-sided Z-Test with unpooled variance was used in the calculation. The significance level of the test was 0.05 was a power of 90%. The minimal sample size was 121 patients in each group. As propensity score matching (PSM) was supposed to be used in this study, we increased the sample size by an additional 25%. Therefore, the final sample size of the study was 302.

### Statistical analysis

Normal distribution was tested by the Kolmogorov–Smirnov test. Continuous data that conformed to a normal distribution were presented as means ± standard deviations. Continuous data that did not conform to the normal distribution were expressed as median (quartile spacing). Categorical data were presented as frequencies (percentages). Continuous data that conformed to a normal distribution were compared using a t-test. Continuous data that did not conform to the normal distribution were compared using the Mann–Whitney test. Categorical data were compared using the chi-square test. A PSM was performed to reduce the bias of confounders. Individual propensity scores were calculated using multivariable logistic regression model accounting for age, gender, the use of laparoscopes, and the use of epidural anesthesia. A 1-to-1 matching was performed to the nearest neighbor methods on the propensity scores with a caliper size of 0.02. The relative ratio (RR) for the incidence of shoulder pain was presented with its confidence interval (CI). The significance threshold was set at 0.05 in all analyses. All statistical analyses were performed using the SPSS (Version 28.0, USA).

## Results

### Flow chart of the study

**Figure 1:**
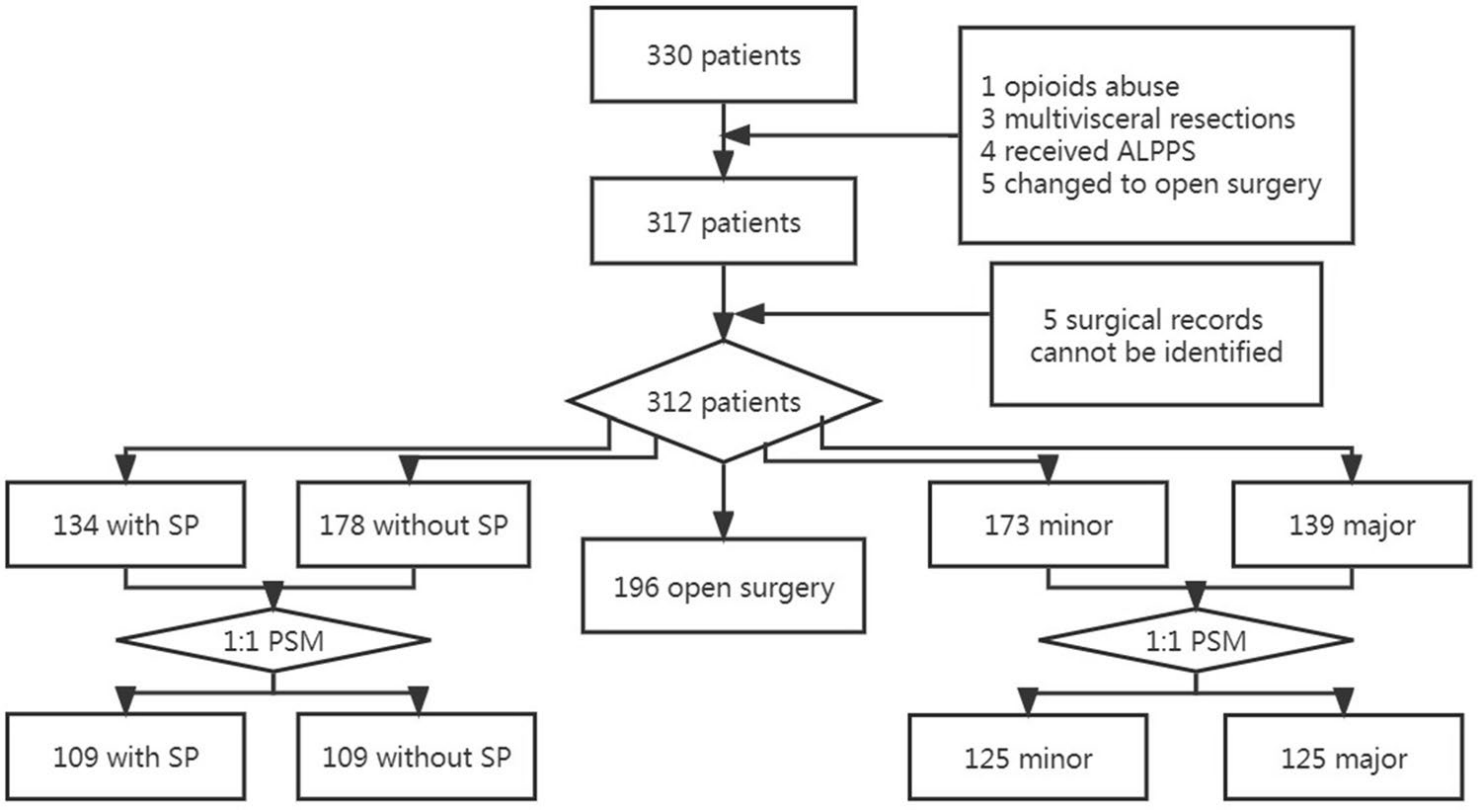
The flow chart of the study. *ALPPS* associating liver partition and portal vein ligation for staged hepatectomy, *SP* shoulder pain, *PSM* propensity score matching.

A total of 330 patients underwent hepatic surgeries from Jun. 2022 to Oct. 2022. According to the exclusion criteria, 13 patients were excluded (1 patient for opioids abuse, 3 patients for receiving multivisceral resections (except gall bladder); 4 patients for receiving associating liver partition and portal vein ligation for staged hepatectomy, 5 patients for changing to open surgery). Five patients were additionally excluded because their surgical records were unidentifiable. Finally, 312 patients were included in the study. The flow chart was shown in Fig. 1.

### The number of surgical segments

Among 312 patients, 134 (42.9%) patients reported shoulder pain within 2 days after surgery. None of the patients reported a relationship between shoulder pain and breathing (or eating). Patients with shoulder pain were significantly younger, and epidural anesthesia was more frequently used among them. After PSM, 109 patients were matched in each group. There were no significant differences in age, gender, BMI, use of laparoscopes, use of epidural anesthesia, bile leakage, surgical duration, and surgical position between the two groups. The drainage volume in the case group was significantly more than that in the control group (*P* = 0.017). A greater number of surgical segments was observed in patients with shoulder pain (*P* = 0.09, Table 1). However, the difference did not reach statistical significance.

**Table 1:** Comparison of surgical segments and basic data before and after PSM.

	Before PSM		After PSM	
	SP (n = 134)	No SP (n = 178)	SP (n = 109)	No SP (n = 109)
Age (per year)	58 (47, 66)	60 (53, 69)**	60 (51, 67)	58 (53, 67)
Gender (male)	84 (60.7%)	123 (69.1%)	76 (69.7%)	76 (69.7%)
BMI (kg/m^2^)	23.4 ± 3.6	23.6 ± 3.6	23.4 ± 3.5	23.6 ± 3.6
Hypertension	23 (17.2%)	36 (20.2%)	23 (21.1%)	25 (22.9%)
Diabetes	10 (7.5%)	17 (9.6%)	9 (8.3%)	11 (10.1%)
Laparoscope	55(41%)	61 (34.3%)	41 (37.6%)	42 (38.5%)
Epidural Anesthesia	87 (64.9%)	90 (50.6%)*	67 (61.5%)	67 (61.5%)
Sufentanil (ug)	30 (20, 35)	30 (20, 40)	30 (20, 35)	30 (20, 40)
Surgical pain	1 (0.75, 2)	1 (1, 2)	1 (1, 2)	1 (1, 2)
Surgical duration	110 (77, 141)	111 (77, 141)	106 (80, 133)	110 (77, 147)
Surgical segments	2 (1, 4)	2 (2, 4)	3 (2, 4)	2 (1, 4)
Surgical position (with cushion)	106 (79.1%)	126 (70.8%)	86 (78.9%)	82 (75.2%)
Drainage volume (ml)	350 (208, 605)	270 (168, 553)	350 (210, 625)	250 (165, 450)*
Bile leakage	4 (3%)	8 (4.5%)	1 (0.9%)	2 (1.8%)

Data were expressed as means ± standard deviations, median (quartile), and N (%).

*PSM* propensity score matching, *SP* shoulder pain. Drainage volume was defined as the drainage volume within 2 days after surgery.

*P < 0.05, **P < 0.01.

### Minor and major hepatectomy

Without PSM, 312 patients were divided into the minor group (n = 173) and the major group (n = 139). In the minor group, laparoscopes were more frequently used (*P* < 0.001, Table 2) and the surgical duration was shorter (*P* < 0.001). There were no differences in the incidence of shoulder pain between the two groups. After PSM, 125 patients were enrolled in each group. There were no differences in those variables except surgical duration (*P* < 0.001) and drainage volume (*P* = 0.016) between these two groups. The incidences of shoulder pain and moderate to severe shoulder pain between the two groups did not reach statistical differences. (*P* = 0.37 and *P* = 0.54).

**Table 2:** Comparison of incidence of shoulder pain between minor and major hepatectomy.

	Before PSM		After PSM	
	Segments ≤ 2 (n = 173)	Segments > 2 (n = 139)	Segments ≤ 2 (n = 125)	Segments > 2 (n = 125)
Age (per year)	59 (51, 67)	60 (53, 68)	60 (52, 69)	60 (52, 69)
Gender (male)	118 (68.2%)	89 (64%)	84 (67.2%)	82 (65.6%)
BMI (kg/m^2^)	23.6 ± 3.5	23.5 ± 3.7	23.6 ± 3.4	23.3 ± 3.7
Hypertension	28 (16.2%)	31 (22.3%)	23 (18.4%)	29 (23.2%)
Diabetes	11 (6.4%)	16 (11.5%)	7 (5.6%)	13 (10.4%)
Laparoscope	82 (47.4%)	34 (24.5%) ***	34 (27.2%)	34 (27.2%)
Epidural anesthesia	93 (53.8%)	84 (60.4%)	81 (64.8%)	76 (60.8%)
Sufentanil (ug)	30 (20, 40)	30 (20, 40)	30 (20, 35)	30 (20, 40)
Surgical pain	1 (1, 2)	1 (1, 2)	1 (1, 2)	1 (1, 2)
Surgical duration	92 (60, 120)	120 (90, 150)***	100 (70, 130)	120 (92, 150)***
Drainage volume (ml)	260 (170, 455)	400 (200, 690)**	270 (175, 490)	410 (210, 685)*
Bile leakage	4 (2.3%)	8 (5.8%)	3 (2.4%)	6 (4.8%)
SP	71 (41%)	63 (45.3%)	49 (39.2%)	57 (45.6%)
MSP	37 (21.4%)	33 (23.7%)	24 (19.2%)	29 (23.2%)

Data were expressed as means ± standard deviations, median (quartile), and N (%).

*SP* shoulder pain, *MSP* moderate to severe shoulder pain. Minor hepatectomy was defined as the removal of one or two liver segments.

*P < 0.05, **P < 0.01, ***P < 0.001.

### The influence of surgical lobes

**Figure 2:**
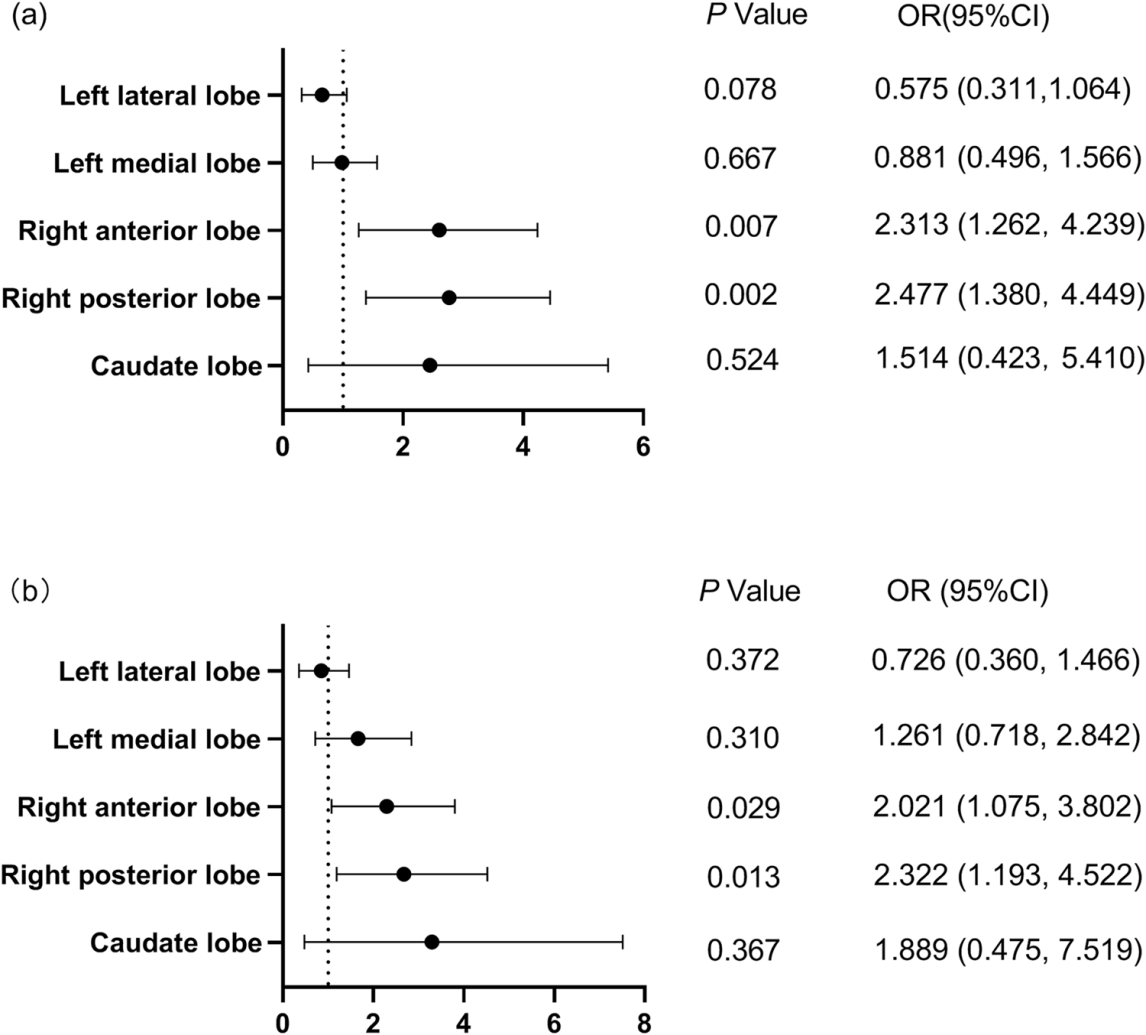
Binary regression analyses for shoulder pain. Univariate logistic regression (**a**) and multivariate logistic regression (**b**) were used to calculate the risk of shoulder pain. The risk of each variable was shown in the odds ratio and its 95% confidence interval. The odds ratio greater than 1 (dotted line) represented an increased risk of shoulder pain.

In patients undergoing open surgeries, the incidence of shoulder pain was 40.3% (79/196). The baseline data of patients were shown in Table 3. Patients with shoulder pain were younger than those without pain. There were no significant differences in the drainage volume between the two groups (*P* = 0.262). Univariate logistic regression analysis showed that the surgical site involving the right anterior lobe (OR (95% CI) 2.313 (1.262, 4.239), *P* = 0.007) and right posterior lobe (OR (95% CI) 2.477 (1.380, 4.449), *P* = 0.002) increased the incidence of shoulder pain. Multivariate logistic regression analysis showed that, from the perspective of surgical procedures, surgical sites involving the right anterior lobe (OR (95% CI) 2.021 (1.075, 3.802), *P* = 0.029) and right posterior lobe (OR (95% CI) 2.322 (1.193, 4.522), *P* = 0.013) were both independent risk factors for shoulder pain (Shown in Fig. 2).

**Table 3:** Demographic characteristics and perioperative data in patients undergoing open surgeries.

	Patients with SP (n = 79)	Patients without SP (n = 117)
Age (per year)	57 (49, 66)	61 (53, 69)*
Gender (male)	54(68.4%)	83(70.9%)
BMI (kg/m^2^)	23.7 ± 3.5	23.6 ± 3.7
Epidural anesthesia	55 (69.6%)	71 (60.7%)
Sufentanil (ug)	30 (20, 30)	30 (20, 40)
Surgical pain	1 (1, 2)	1 (1, 2)
Surgical duration (min)	110 (82, 149)	120 (88, 148)
Drainage volume (ml)	430 (220, 630)	300 (170, 610)
Bile leakage	2 (2.5%)	7 (6%)

Data were expressed as means ± standard deviations, median (quartile), and N (%).

*SP* shoulder pain.

*P < 0.05.

A total of 21 patients received open right hemihepatectomy. Among them, 10 (47.6%) patients reported shoulder pain.

### The site of shoulder pain

**Figure 3:**
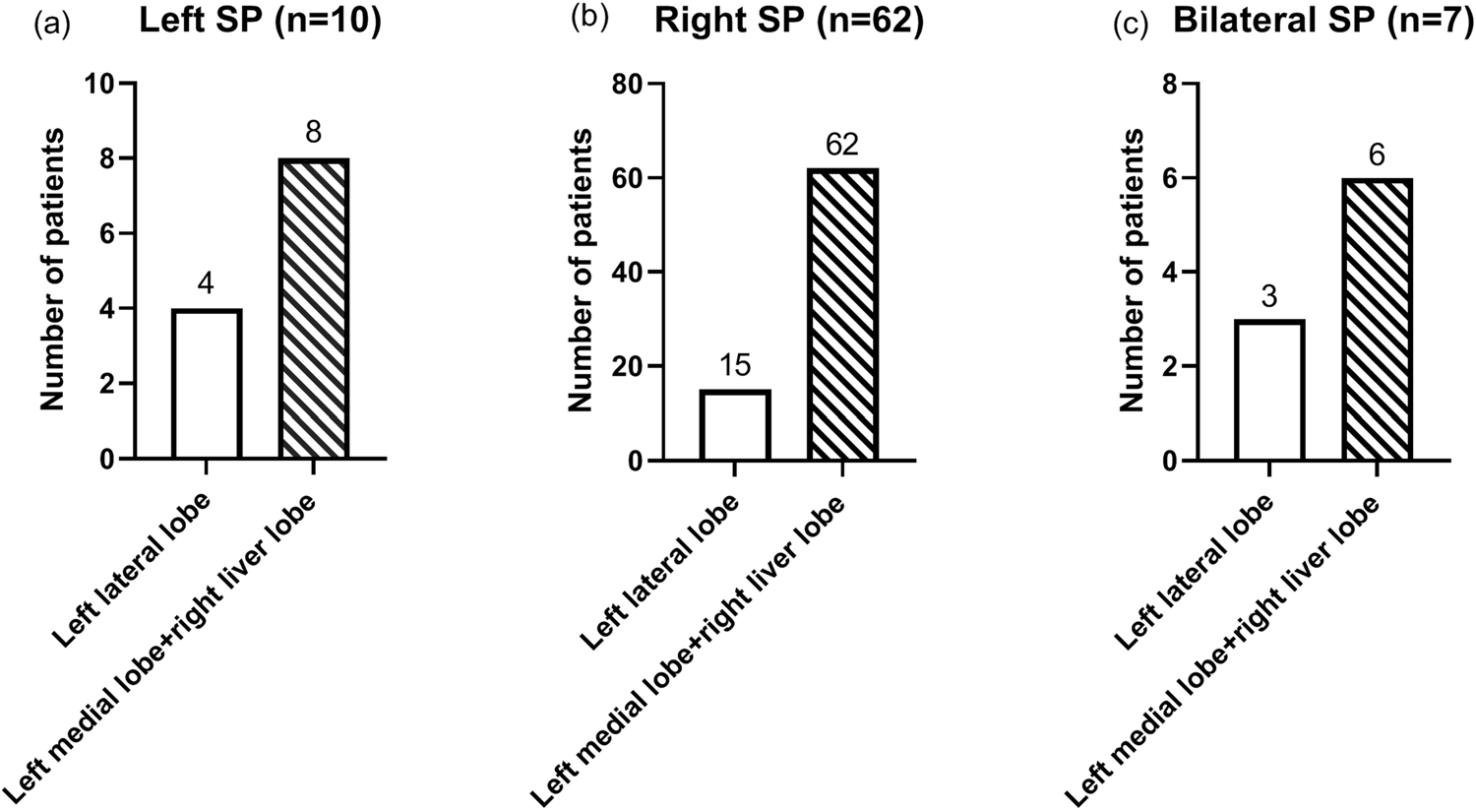
The site of shoulder pain and surgical sites. Patients were divided into three subgroups according to the sites of shoulder pain. The surgical sites of each patient were recorded in these three groups, respectively. Left lateral lobe: surgical sites involving the left lateral lobe. Left medial lobe + right liver lobe: surgical sites involving the left medial lobe or right liver lobe.

The data on the site of pain was shown in Fig. 3. In patients undergoing open surgeries, 10 patients reported left shoulder pain, 62 patients reported right shoulder pain, and 7 patients reported bilateral shoulder pain. In patients with left shoulder pain, 4 (40%) patients received liver resections involving the left lateral lobe. In patients with right shoulder pain, 62 (100%) patients received liver resections involving the right liver lobe or (and) left medial lobe.

## Discussion

In this cohort study, we found that minor hepatectomy did not reduce the incidence of PHSP. Furthermore, the nested case–control analyses showed that hepatectomy involving the right lobe of the liver was the independent risk factor for PHSP.

The case–control analysis showed the tendency that more liver segments were resected in the patients who reported shoulder pain. However, the differences were relatively slight and not statistically significant. Minor hepatectomy was defined as the removal of one or two liver segments^9^. In the comparison of these two groups, minor hepatectomy showed a similar incidence of PHSP. The possible explanation was that even in minor hepatectomy, for a better surgical view, the superior liver border was also mobilized from the diaphragm. The tissue damage during the separation may irritate the diaphragm, which induces PHSP. The comparison of the incidence of shoulder pain showed slight differences before and after PSM, mainly because laparoscopes were performed more frequently in minor hepatectomies. The pneumoperitoneum produced by the laparoscopy can directly cause postoperative shoulder pain in many types of surgeries^10,11^. In clinical practice, to reduce surgical trauma, surgeons tend to perform minor hepatectomy using a laparoscopic technique (verified in this study). As a result, minor hepatectomy may cause a higher incidence of PHSP in the clinical observation. Therefore, we suggested that the risk of shoulder pain after minor hepatectomy should be equally valued during the perioperative period.

As pneumoperitoneum alone may also cause shoulder pain, in the analysis of the risk of specific liver lobes, we excluded laparoscopic surgeries to reduce the bias. In the case–control analysis, we found that surgical sites involved with the right anterior and right posterior lobes were both independent risk factors for PHSP. The most likely explanation was that the right liver lobe was more closely adjacent to the diaphragm. The intraoperative mechanical stimulus directly irritates the diaphragm. Furthermore, the subsequent inflammatory responses around the diaphragm may also contribute to the maintenance of shoulder pain^11^. In this study, we found that the drainage volume was significantly more in those patients with shoulder pain, which implied that postoperative hemorrhage may irritate the diaphragm and increase the risk of shoulder pain. However, significant differences were not found in the patients undergoing open surgeries. The possible reasons were: (1) Shoulder pain can be caused by a variety of causes. (2) The sample size was relatively small after we exclude laparoscopic surgeries.

Head et al.^12^ found that when hepatic tumors were located adjacent to the diaphragm, radiofrequency ablation caused referred shoulder pain. In addition, most of these patients were found diaphragmatic injuries after CT scans. Lguchi et al*.*^13^ also found that in radiofrequency ablation of pulmonary tumors, the patients with tumors near the diaphragm showed higher incidences of shoulder pain. However, in those studies, thermal injury was the primary cause of diaphragmatic injury. Whether intraoperative procedures showed a similar effect has not been verified in previous studies. Another possible explanation was the operative position. The special operative position of the right hepatectomy may overly abduct the shoulder joint. Olumuyiwa et al.^14^ pointed out that ligament strain and referred phrenic nerve pain contributed to post-operative shoulder pain. However, Miyoshi et al. found that most patients reported right shoulder pain even in a symmetrical position during hepatectomy, which indicated that PHSP is not a type of myofascial pain^4^. Based on the case–control analysis, we found that the surgical position in our institution did not increase the risk of shoulder pain. Whereas, in the left lateral lobe hepatectomy, the left hepatic vein was relatively easy to expose, which reduce the irritation of the diaphragm during the process. These findings suggested that stronger prevention strategies should be applied in the patients who receive the right hepatectomies.

The treatments of referred shoulder pain can be classified as interventions on pneumoperitoneum, pharmacological treatments, and invasive treatment. Among invasive treatments, PNB was the most frequently used therapy. Bak et al.^3^ have reported that PNB was effective in the treatment of PHSP. Previous studies pointed out that PNB during the perioperative period can reduce the incidence of shoulder pain after thoracic surgery and laparoscopic cholecystectomy^15–17^. In this study, we found for the first time that left shoulder pain may also occur in those patients who received left medial lobe or right hepatectomy. The left lateral lobe of the liver lies to the left of the medial axis of the diaphragm. These findings indicated that the location of the surgical site did not determine the site of shoulder pain, which suggested that ipsilateral prophylactic nerve block may not be suitable for the prevention of PHSP. This phenomenon may be explained by the hypothesis that during open right hepatectomy, the inferior vena cava serves as a fulcrum. When the right liver is pulled down, it may cause the left lateral lobe to rise and irritate the diaphragm, which ultimately results in left shoulder pain.

The difficulty in prophylactic nerve block indicated that pharmacological intervention may be a future direction for the prevention of PHSP. Past studies found that neither pregabalin nor gabapentin was effective in the prevention of referred shoulder pain^18,19^. In addition, the effectiveness of opioids in the prevention of referred shoulder pain was controversial^4,20^. In the nested case–control analysis, we found that there were no differences in the amount of intraoperative sufentanil between the two groups, which is contrary to the previous study^4^. Our finding suggested that intraoperative opioids cannot reduce the risk of PHSP. Intraoperative high-dose opioid therapy may increase the risk of postoperative nausea and vomiting^21^, which was not an ideal means of the prevention of PHSP. Therefore, additional trials were needed to investigate the effect of analgesic drugs in preventing shoulder pain.

There were a few limitations in this study. Firstly, specific types of hepatectomies were not included in this study, because it is difficult to categorize liver resections strictly. Secondly, it was a single-center study. All operations were performed by the same group of surgeons. Similar surgical manipulations may cause sample bias. In addition, the surgical positions in our hospital may be different from other institutions. Therefore, the findings in this study need to be further confirmed in future studies in other institutions.

## Conclusion

In conclusion, minor hepatectomy also showed a high incidence of postoperative shoulder pain. Surgical procedures on the right anterior lobe and right posterior lobe were both independent risk factors for shoulder pain. Stronger prevention measures should be used in those high-risk patients.

## Supplementary Information

Supplementary Figure 1.

Supplementary Legends.

## Data Availability

The datasets used during the current study are available from the corresponding author upon reasonable request.
