# Supplementary figures and images for: Influence of extent of surgical resection on post-hepatectomy shoulder pain: an observational study
Source: Sci Rep. 2023 Jul 5;13:10861. doi: 10.1038/s41598-023-38052-6 (PMC10322930; doi:10.1038/s41598-023-38052-6)

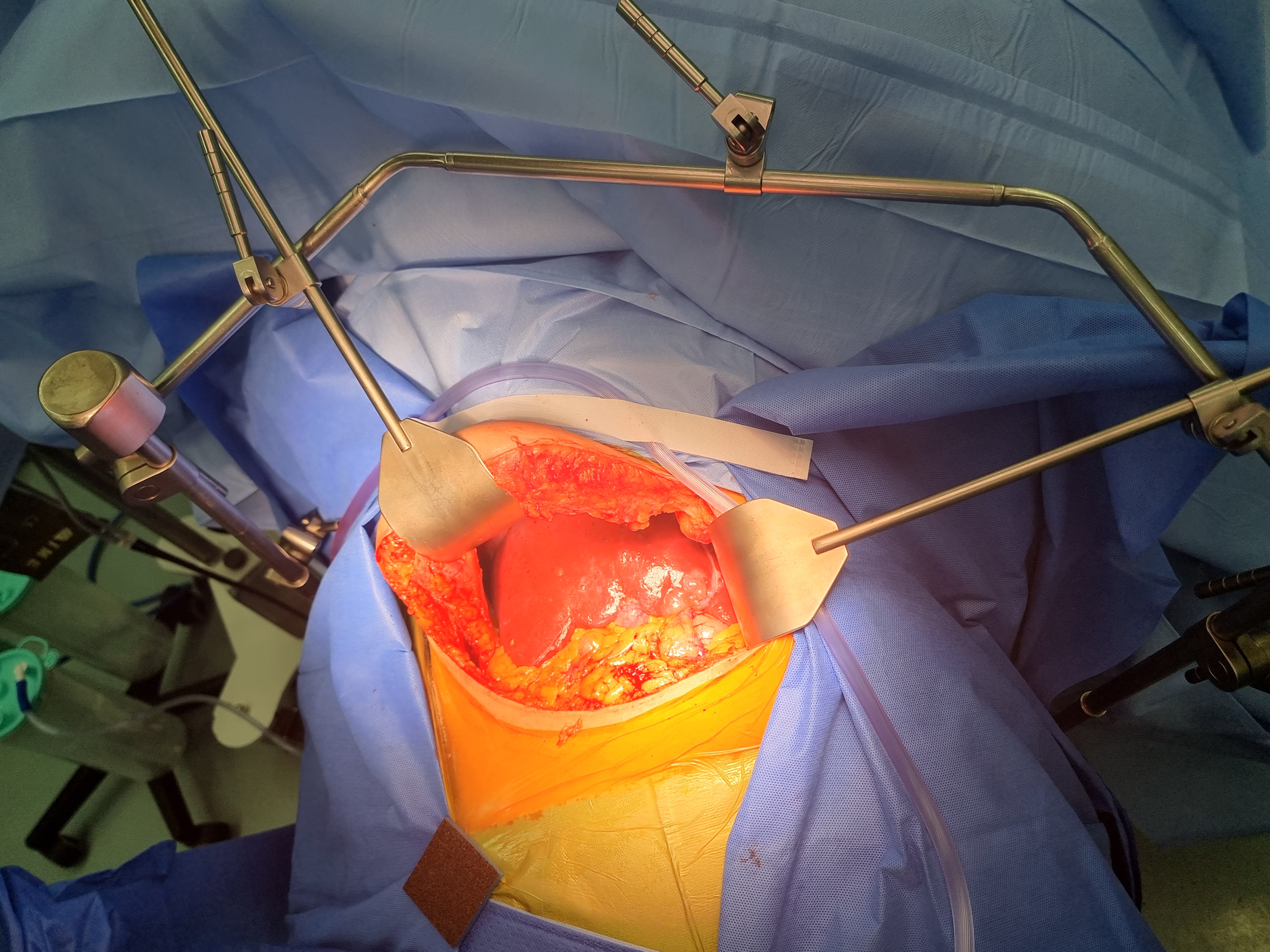

Supplement: Supplementary file 1 — Supplementary Figure 1. [file 41598_2023_38052_MOESM1_ESM.jpg]
