# Supplementary material for: Influence of extent of surgical resection on post-hepatectomy shoulder pain: an observational study
Source: Sci Rep. 2023 Jul 5;13:10861. doi: 10.1038/s41598-023-38052-6 (PMC10322930; doi:10.1038/s41598-023-38052-6)
Supplement: Supplementary file 2 — Supplementary Legends. [file 41598_2023_38052_MOESM2_ESM.docx]

Figure legend: All incisions were made below the right costal margin. Retractors (Omni-Tract) were used to expose the operative field.
